# Expression of nephronectin is enhanced by 1α,25‐dihydroxyvitamin D_3_

**DOI:** 10.1002/2211-5463.12085

**Published:** 2016-07-13

**Authors:** Katsuhiro Hiranuma, Atsushi Yamada, Tamaki Kurosawa, Ryo Aizawa, Dai Suzuki, Yoshiro Saito, Ryo Nagahama, Mikiko Ikehata, Masayuki Tsukasaki, Naoko Morimura, Daichi Chikazu, Koutaro Maki, Tatsuo Shirota, Masamichi Takami, Matsuo Yamamoto, Takehiko Iijima, Ryutaro Kamijo

**Affiliations:** ^1^ Department of BiochemistrySchool of DentistryShowa UniversityTokyoJapan; ^2^ Department of Perioperative MedicineDivision of AnesthesiologySchool of DentistryShowa UniversityTokyoJapan; ^3^ Department of PeriodontologySchool of DentistryShowa UniversityTokyoJapan; ^4^ Department of Oral and Maxillofacial SurgerySchool of DentistryShowa UniversityTokyoJapan; ^5^ Department of OrthodonticsSchool of DentistryShowa UniversityTokyoJapan; ^6^ Department of Oral and Maxillofacial SurgeryTokyo Medical UniversityJapan; ^7^ Brain Science LaboratoryThe Research Organization of Science and TechnologyRitsumeikan UniversityKusatsuShigaJapan; ^8^ Department of PharmacologySchool of DentistryShowa UniversityTokyoJapan; ^9^ Present address: Department of ImmunologyGraduate School of Medicine and Faculty of MedicineThe University of TokyoHongo 7‐3‐1, Bunkyo‐kuTokyo113‐0033Japan

**Keywords:** 1α,25‐dihydroxyvitamin D_3_, nephronectin, vitamin D receptor

## Abstract

The extracellular matrix protein nephronectin (Npnt), also called POEM, is considered to play critical roles as an adhesion molecule in development and functions of various tissues, such as the kidneys, liver, and bone. In the present study, we examined the molecular mechanism of Npnt gene expression and found that vitamin D_3_ (1α,25‐dihydroxyvitamin D_3_,VD_3_) strongly enhanced Npnt mRNA expression in MC3T3‐E1 cells from a mouse osteoblastic cell line. The VD_3_‐induced increase in Npnt expression is both time‐ and dose‐dependent and is mediated by the vitamin D receptor (VDR).

**Abbreviations**
JAK: janus kinase
MAM: meprin A5 protein and receptor protein‐tyrosine phosphatase μ
MAPK: mitogen‐activated protein kinase
Npnt: nephronectin
RANKL: receptor activator of nuclear factor kappa‐β ligand
RGD: Arg‐Gly‐Asp
RXR: retinoic acid receptor
STAT: signal transducer and activator of transcription
TGF‐β: transforming growth factor‐β
TNF‐α: tumor necrosis factor‐α
VD_3_: 1α,25‐dihydroxyvitamin D_3_
VDRE: vitamin D response elements
VDR: vitamin D receptor

Nephronectin (Npnt), an extracellular matrix protein, is known to be involved in the development and functions of various tissues 1, 2. This protein, which acts as an adhesion molecule, consists of five epidermal growth factor (EGF)‐like domains, an Arg‐Gly‐Asp (RGD) cell binding motif, and a meprin A5 protein and receptor protein‐tyrosine phosphatase μ (MAM) domain 1, 2. Npnt interacts with integrins, especially α8β1 integrins, and plays a crucial role during the early steps of kidney morphogenesis through its own RGD motif 2. Ablation of Npnt was reported to induce kidney agenesis or hypoplasia, as well as delocalization of the arrector pili muscle associated with the hair follicle bulge in the epidermis 3, 4.

The active form of vitamin D_3_ (1α,25‐dihydroxyvitamin D_3_; VD_3_) plays a crucial role in regulating calcium and phosphate homeostasis in several target tissues including the kidneys, small intestine, and bone 5. The functions of VD_3_ in bone include both stimulation and inhibition of mineralization, and *in vitro* studies have revealed that VD_3_ regulates osteoblast‐lineage cell growth and function depending on cell source and the initial state of differentiation 5, 6. On the other hand, VD_3_ also regulates the expression of receptor activator of nuclear factor‐κβ ligand (RANKL), which stimulates bone resorption through osteoclast activation 7. The biological functions of VD_3_ are mediated by its binding to the vitamin D receptor (VDR), a nuclear transcription factor that forms a heterodimer with the retinoid X receptor (RXR) and binds to vitamin D responsible elements (VDRE) in regulatory regions that are functionally linked to specific target genes 8, 9.

In our previous studies, we found that Npnt expression is downregulated by cytokines, such as TGF‐β, TNFα, and oncostatin M, via MAPK, JAK/STAT, and NF‐κβ pathways in MC3T3‐E1 cells 10, 11, 12. The present study clearly shows that VD_3_ upregulates the expression of Npnt in both a time‐ and dose‐dependent manner via the VDR.

## Results and Discussion

**Figure 1:**
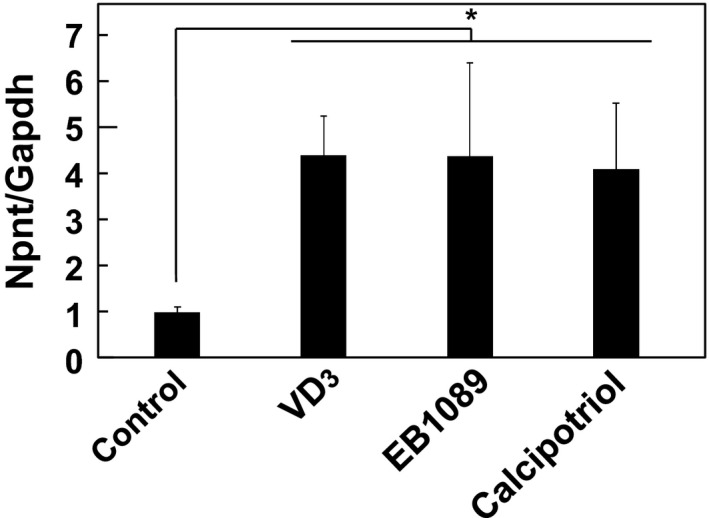
Npnt mRNA expression increased by treatment with VD_3_. MC3T3‐E1 cells were treated with 100 ng·mL^−1^ of VD_3_, EB1089, or calcipotriol for 24 h. Total cellular RNA was extracted, and mRNA levels for Npnt and Gapdh were examined using quantitative real‐time PCR analysis. Results are shown as the mean ± SD of three samples. *P < 0.05, Student's *t* test, relative to the level at 0 ng·mL^−1^.

In this study, we initially examined regulation of the expression of Npnt by VD_3_, as well as by its agonistic analogs EB1089 and calcipotriol, in MC3T3‐E1 cells. Treatment with 100 ng·mL^−1^ of each those reagents for 24 h sharply increased the expression of Npnt mRNA (Fig. 1). In addition, VD_3_ treatment increased Npnt gene expression in C2C12 cells from a mouse myoblast cell line and in STC‐1 cells from a mouse intestinal cell line, whereas no such increase was seen in HEK293 cells from a human embryonic kidney cell line (Fig. S1).

**Figure 2:**
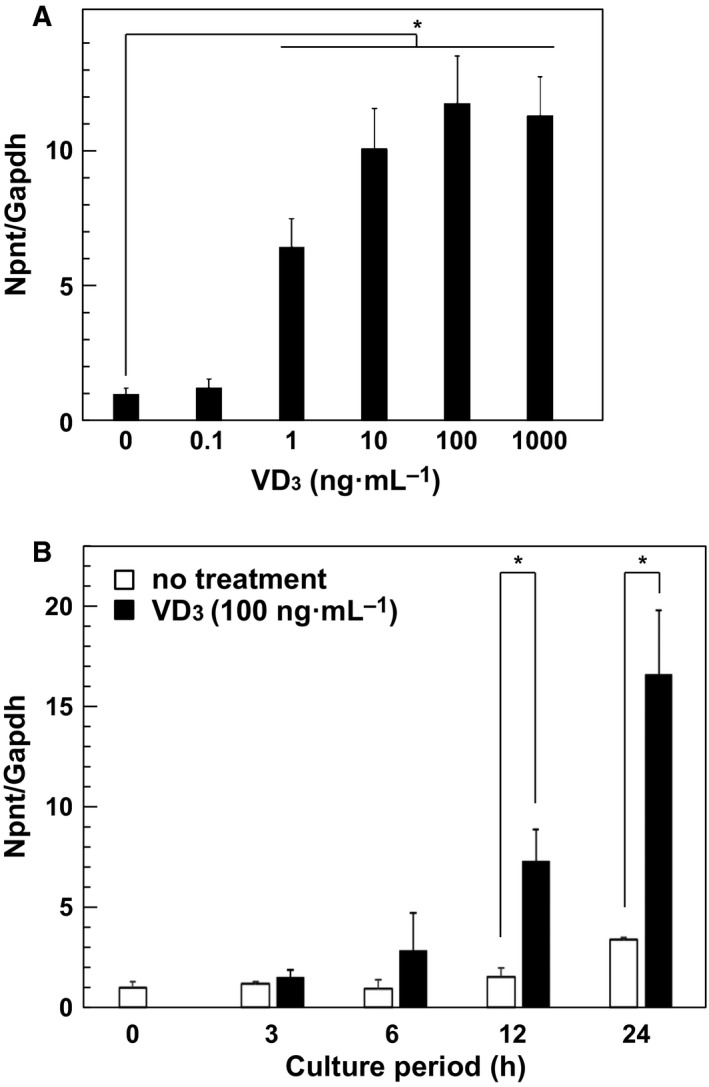
Effects of VD_3_ on Npnt mRNA expression. (A) Dose‐dependent effects of VD_3_ on Npnt mRNA expression. MC3T3‐E1 cells were treated with 0, 0.1, 1, 10, 100, or 1000 ng·mL^−1^ of VD_3_ for 24 h. Total cellular RNA was extracted, and mRNA levels for Npnt and Gapdh were examined using quantitative real‐time PCR analysis. Results are shown as the mean ± SD of three samples. *P < 0.05, Student's *t* test as compared to the level with 0 ng·mL^−1^ of VD_3_. (B) Time‐course analysis of effects of VD_3_ on Npnt mRNA expression. MC3T3‐E1 cells were treated with 100 ng·mL^−1^ of VD_3_ for 3, 6, 12, or 24 h. Results are shown as the mean ± SD from three samples. *P < 0.05, Student's *t* test, relative to the level with 0 ng·mL^−1^ of VD_3_ at each time point.

In the above described experiments, MC3T3‐E1 cells were treated with different concentrations of VD_3_ for 24 h and we observed a significant increase in Npnt mRNA expression, when the VD_3_ concentration was greater than 10 ng·mL^−1^ when compared to the unstimulated control (Fig. 2A). The time‐dependent effects of VD_3_ on Npnt mRNA expression was then further examined using a fixed concentration of 100 ng·mL^−1^. When the cells were treated for at least 12 h, a significant increase in Npnt mRNA expression was seen and occurred in a time‐dependent manner, which increased more than up to 15‐fold after 24 h (Fig. 2B).

**Figure 3:**
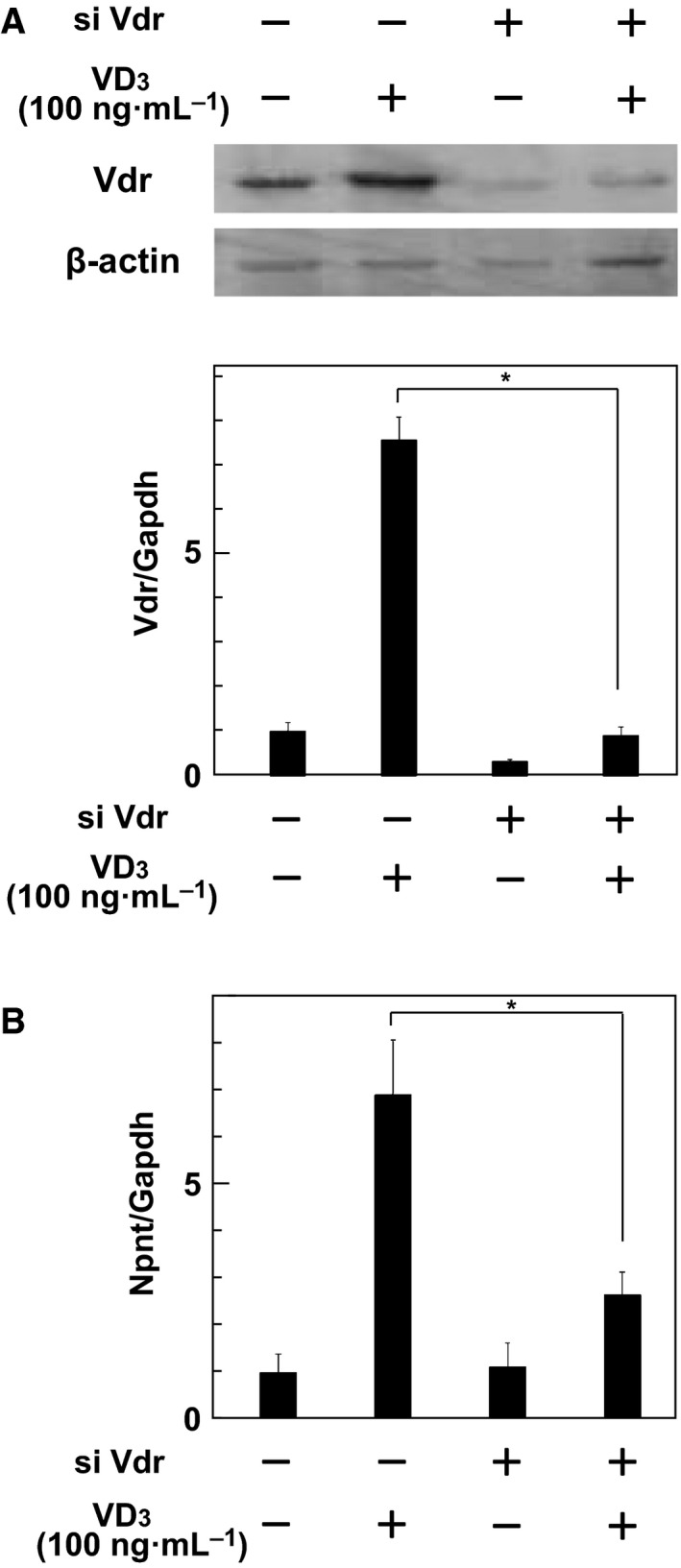
siRNA‐mediated knockdown of the VDR reduced Npnt gene expression in osteoblasts mediated by VD_3_. VDR siRNA was introduced to MC3T3‐E1 cells and incubation was performed for 48 h. After extracting total cellular RNA and protein, mRNA samples for Npnt and Gapdh VDRs were examined using quantitative real‐time PCR, and protein samples for those VDRs and β‐actin were examined by western blotting. (A) VDR expression was suppressed by introducing VDR siRNA. Results are shown as the mean ± SD of three samples. *P < 0.05, Student's *t* test, relative to the level without VDR siRNA. (B) VD_3_‐induced Npnt gene expression was downregulated by VDR siRNA. Results are shown as the mean ± SD of three samples. *P < 0.05, Student's *t* test, relative to the level without VDR siRNA.

To investigate the mechanism that governs regulation of Npnt expression through the VDR in osteoblasts, MC3T3‐E1 cells were treated with a small interfering RNA (siRNA) targeting VDR. First, we noted significant decreases in VDR mRNA and protein levels in MC3T3‐E1 cells after the treatment with VDR siRNA (Fig. 3A), which decreased Npnt gene expression (Fig. 3B), suggesting that Npnt gene expression is regulated by the VDR.

**Figure 4:**
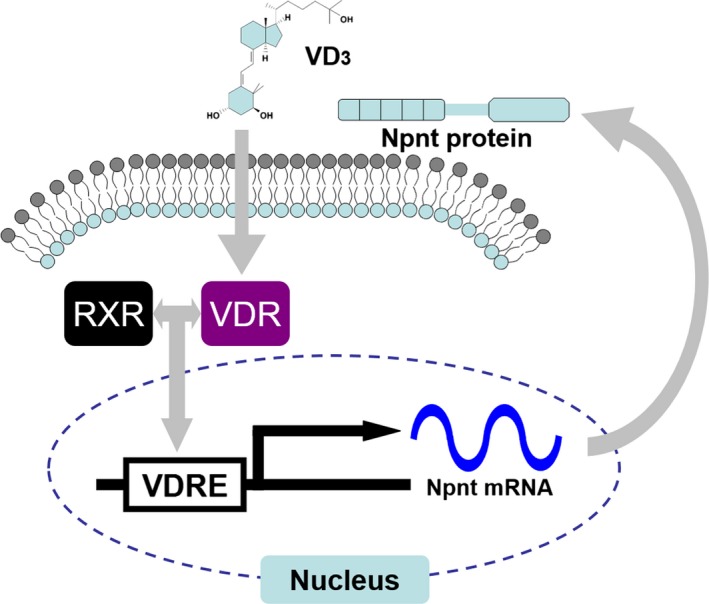
Proposed model of increased Npnt mRNA expression induced by VD_3_ through the VDR. VD_3_ binding to the VDR forms a heterodimer with RXR, which may bind to VDRE in the hypothetical regulatory regions of the Npnt gene to regulate its expression.

On the basis of our results, we propose a model of increased Npnt mRNA expression induced by VD_3_ through the VDR (Fig. 4). VD_3_ binding to the VDR forms a heterodimer with the retinoid X receptor (RXR), which may bind to VDRE in hypothetical regulatory regions of the Npnt gene to regulate its expression. Although Tsukasaki *et al*. 11 hypothesized that putative VDRE can be detected by a transcriptional factor search program (alibaba) and are located upstream of the transcriptional starting site of the Npnt gene (− 133 to − 112 and − 663 to − 642), no evidence of VDRE for the Npnt gene has been detected yet (data not shown).

Kahai *et al*. 13 demonstrated that one of the physiological functions of Npnt is the enhancement of osteoblast differentiation via EGF‐like domains, which activates the ERK‐MAPK signaling pathway. On the other hand, the anabolic and catabolic functions of VD_3_ in bone that occur via osteoblast differentiation are associated with VD_3_ and VDR‐dependent changes in gene expression 5. To determine the relationship between osteoblast differentiation and VD_3_‐induced Npnt gene expression, Npnt siRNA‐treated MC3T3‐E1 cells were treated with/without BMP‐2 or VD_3_. Unexpectedly, VD_3_‐induced Npnt gene induction did not affect osteoblast differentiation of MC3T3‐E1 cells (Fig. S2). Additional studies are required to determine the precise physiological and functional roles of Npnt gene expression by VD_3_.

In conclusion, our results show that VD_3_ stimulates Npnt gene expression in both time‐ and dose‐dependent manners via the VDR.

## Materials and methods

### Cell cultures

MC3T3‐E1 cells were maintained in MEMα with L‐glutamine and phenol red medium (Wako Pure Chemical Industries, Ltd., Osaka, Japan; Cat. No. 135‐15175), supplemented with 10% fetal bovine serum (FBS) (Life Technologies, Rockville, MD, USA; Cat. No. 10437) and 1% penicillin‐streptomycin at 37 °C in a CO_2_ incubator (5% CO_2_, 95% air). For the experiments, cells were plated at 3.8 × 10^5^ cells/well in six‐well plates (Thermo Scientific Inc., Waltham, MA, USA; Cat. No. 140675). C2C12 cells were maintained in DMEM (Wako Pure Chemical Industries, Ltd.; Cat. No. 044‐29765) supplemented with 15% FBS and 1% penicillin–streptomycin at 37 °C in a CO_2_ incubator. HEK293 and STC‐1 cells were maintained in DMEM supplemented with 10% FBS and 1% penicillin–streptomycin at 37 °C in a CO_2_ incubator.

### Reagents

1α,25‐dihydroxyvitamin D_3_ (VD_3_) (Calcitoriol; Cat. No. 71820) was purchased from Cayman Chemical Company, Ann Arbor, MI, USA. EB1089 (Cat. No. 3993) was purchased from Tocris Bioscience (Bristol, UK) and calcipotriol (Cat. No. C4369‐10MG) was purchased from Sigma‐Aldrich 14, and recombinant human bone morphogenetic protein‐2 (BMP‐2) (Cat. No. 355‐BM) was purchased from R&D Systems (Minneapolis, MN, USA).

### Quantitative real‐time PCR

Total RNA was extracted using TRIzol reagent (Life Technologies; Cat. No. 15596018), then reverse transcribed using SuperScript III (Life Technologies; Cat. No. 18080‐044). Quantitative real‐time PCR was performed using a Fast SYBR Green Master Mix (Applied Biosystems, Carlsbad, CA, USA; Cat. No.1411119) with the following specific PCR primers: mouse glyceraldehyde 3‐phosphate dehydrogenase (Gapdh), 5′‐AAATGGTGAAGGTCGGTGTG‐3′ and 5′‐TGAAGGGGTCGTTGATGG‐3′; and mouse nephronectin (Npnt), 5′‐CACGAGTAATTACGGTTGACAACAG‐3′ and 5′‐CTGCCGTGGAATGAACACAT‐3′; human glyceraldehyde 3‐phosphate dehydrogenase (Gapdh), 5′‐GCACCGTCAAGGCTGAGAAC‐3′ and 5′‐TGGTGAAGACGCCAGTGGA‐3′; and human nephronectin (Npnt), 5′‐CCAAATGCTGAGCTCACTGAA‐3′ and 5′‐CACCGCCACACTAGGACATTA‐3′.

Quantitative real‐time PCR assays were also performed using TaqMan Fast Universal PCR Master Mix (Applied Biosystems; Cat. No.1501411) with the following specific assay IDs: Vdr, Mn00437297; and Gapdh, Mn03302249g1.

### siRNA knockdown of gene expression

MC3T3‐E1 cells at 50% confluence were seeded and transfected with 10 pmol·cm^−2^ of the culture surface area of the siRNA pool (Stealth siRNA; Life Technologies) using Lipofectamine RNAi MAX reagent (Cat. No.1662469; Life Technologies) in OPTI‐MEM (Cat. No. 31985‐070; Life Technologies). The following IDs were used for Vdr siRNAs; MSS238646, MSS238647, and MSS238682, and those for Npnt siRNAs; MSS272952, MSS272953, and MSS272954.

### Western blotting

Protein samples were collected using Sample Buffer Solution with Reducing Reagent (6×) for SDS/PAGE (Nacalai Tesque, Kyoto, Japan; Cat. No. 09499‐14) with a cell scraper (Corning Incorporated, Corning, NY, USA; Cat. No. 3010), then electrophoresed onto a 10% SDS polyacrylamide gel and blotted onto a PVDF membrane. The PVDF membrane was soaked in TBST solution for 24 h at 4 °C, after which the blots were incubated with primary (dilution; 1 : 100) and secondary (dilution; 1 : 100) antibodies for 1 h. The primary antibody for VDR (Cat. No.sc‐13133) was purchased from Santa Cruz Biotechnology, Inc. (Santa Cruz, CA, USA; Cat. No.sc‐13133). The secondary antibody was an anti‐(mouse IgG) horseradish peroxidase linked antibody and purchased from GE Healthcare (Chicago, IL, USA; Cat. No. NA931VS). To visualize the locations of the antigenic bands, peroxidase reactions were developed using ECL prime western blotting detection reagent (GE Healthcare).

### Alkaline phosphatase activity

Alkaline phosphatase (ALP) activity was determined as a marker of osteoblast differentiation. After removing culture medium, cell layers were washed with PBS, then sonicated in 50 mm Tris‐HCl (pH 7.5) containing 0.1% Triton X‐100. ALP activity in the lysates was determined following incubation with the substrate, *p*‐nitrophenylphosphate, in buffer (pH 10) containing 0.1 m 2‐amino‐2‐methyl‐1‐propanol and 2 mm MgCl_2_. The reaction was terminated by adding NaOH and values were determined at 405 nm.

## Author contributions

AY, RA, MT, NM, DC, KM, TS, MT, MY, TI and RK conceived and designed the experiments. KH, AY and TK performed the experiments. KH, AY and RK analyzed the data. DS, YS, RN and MI contributed reagents/materials/analysis tools. KH, AY and RK wrote the manuscript.

## Supporting information


**Fig. S1.** Dose‐dependent effects of VD_3_ on Npnt mRNA expression in (A) C2C12, (B) HEK293, and (C) STC‐1 cells after treatment with 0, 10, 100, or 1000 ng·mL^−1^ for 24 h.

**Fig. S2.** VD_3_‐induced Npnt gene induction does not affect osteoblast differentiation of MC3T3‐E1 cells.
