# Supplementary figures and images for: Expression of nephronectin is enhanced by 1α,25‐dihydroxyvitamin D3
Source: FEBS Open Bio. 2016 Jul 13;6(9):914–8. doi: 10.1002/2211-5463.12085 (PMC5011489; doi:10.1002/2211-5463.12085)

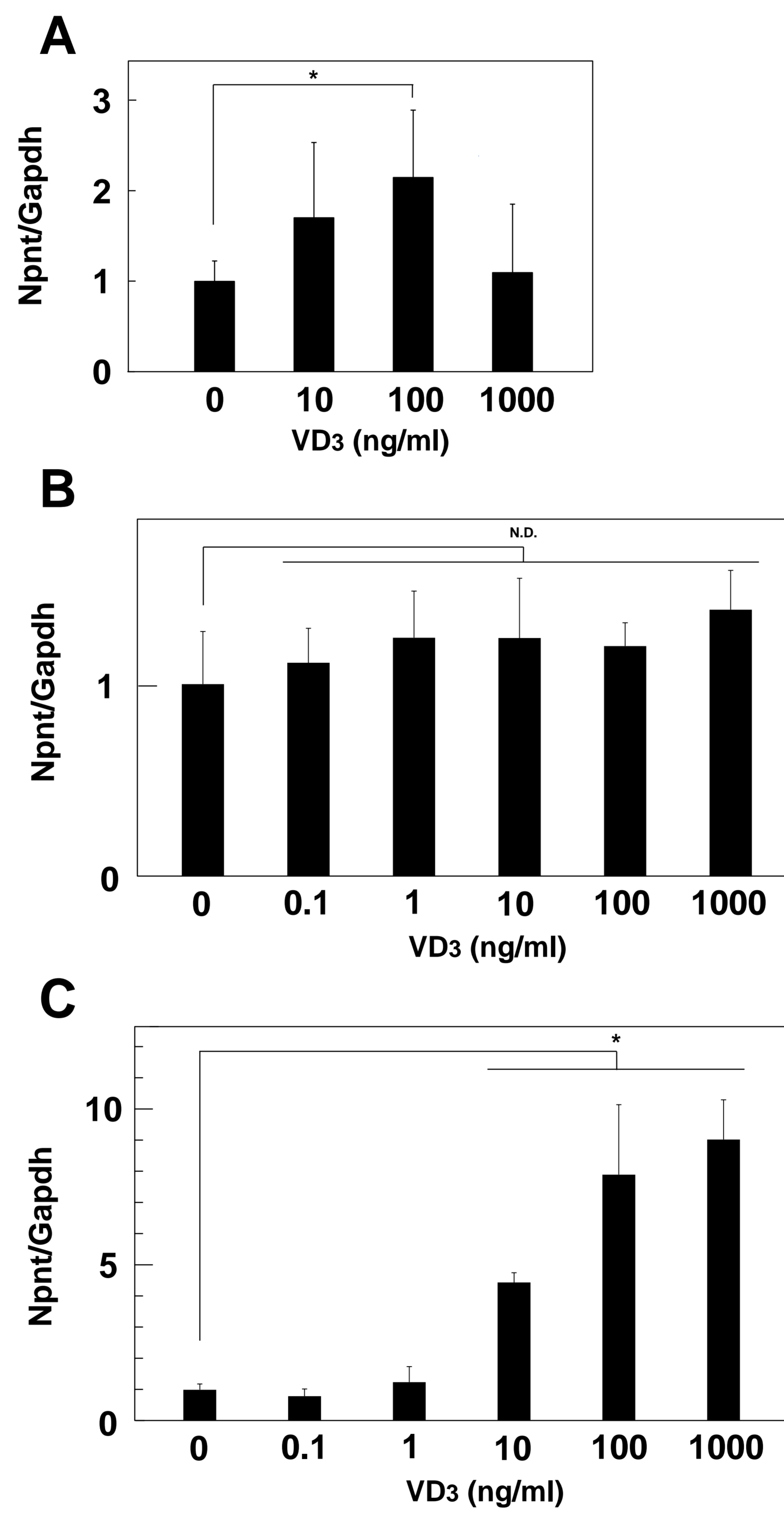

Supplement: Supplementary file 1 — Fig. S1. Dose‐dependent effects of VD3 on Npnt mRNA expression in (A) C2C12, (B) HEK293, and (C) STC‐1 cells after treatment with 0, 10, 100, or 1000 ng·mL−1 for 24 h. [file FEB4-6-914-s001.pdf]

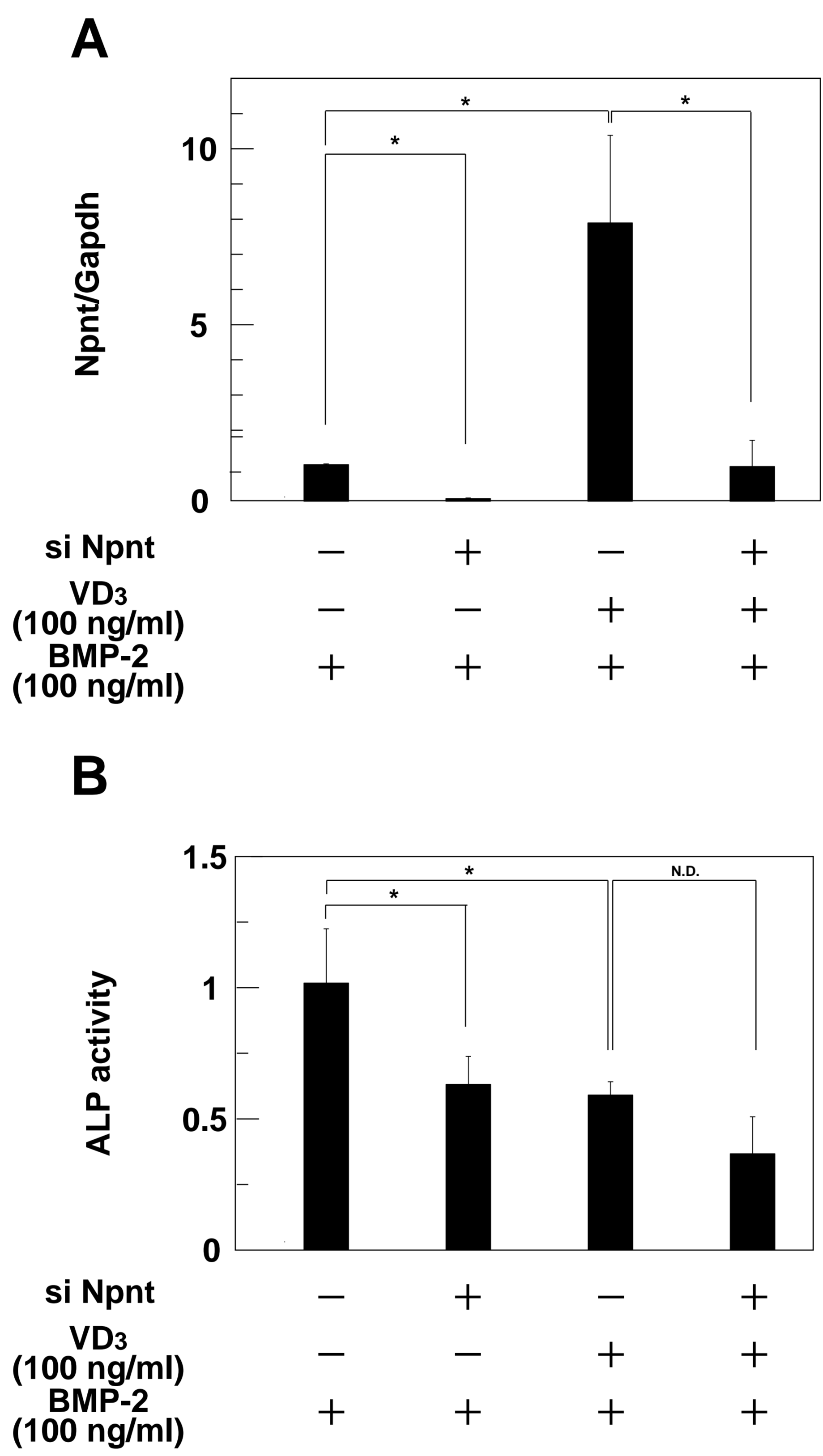

Supplement: Supplementary file 2 — Fig. S2. VD3‐induced Npnt gene induction does not affect osteoblast differentiation of MC3T3‐E1 cells. [file FEB4-6-914-s002.pdf]
